# Complex histopathological and surgical aspects in a case of giant malignant gastric perforation

**Published:** 2016

**Authors:** D Serban, C Branescu, C Savlovschi, A El-Khatib, C Tudor, A Nica, A Kraft, AM Dascalu

**Affiliations:** * “Carol Davila” University of Medicine and Pharmacy, Bucharest, Romania; ** University Emergency Hospital, Upper Digestive Surgery Clinic, Bucharest, Romania

**Keywords:** mucinous gastric cancer, perforation, surgery, omentoplasty

## Abstract

We present the case of a 52-year-old male patient, hospitalized on an emergency basis in the University Emergency Hospital in Bucharest, after being diagnosed with pneumoperitoneum acute abdomen, for which emergency surgery was mandatory. A 3,5-4 cm malignant gastric perforation, ascitis and peritoneal carcinomatosis were found. The histopathological exam revealed infiltrative mucinous gastric carcinoma with epiploic metastasis. Due to the lack of available gastric material, an atypical surgical solution was performed: gastric packing with epiploic material by means of transgastric traction.

The solution proved to be successful for short-term recovery. The underlying condition was not focused on, the patient being directed to the Oncology Department.

Acute gastric perforation is a rare complication of gastric cancer, and the association with gastric linitis is uncommon. This specific histopathological condition made the classical surgical repair techniques unsuitable for the presented case and an atypical solution had to be performed.

## Introduction

Perforated gastric cancer is a rare condition with a reported incidence of less than 1%, and, generally, present with histories and symptoms that do not obviously differ from those of benign gastric perforation. In a review of literature, the ideal operation for treating the malignancy is still in discussion, as it is dependent on various factors such as the hemodynamic stability of the patient, the surgical expertise, the type and stage of the malignancy [**1**]-**3**]. Even if gastric cancer has been diagnosed pre- or perioperatively, it may still be difficult to assess the true extent of the carcinoma and to determine the local operability, due to the inflammatory changes associated with peritonitis, that can affect both the gastric walls and the abdominal lymph-nodes [**3**],**5**]. 

Based on these facts, a two-stage operation is considered by some authors the correct attitude in perforated gastric cancer. In most cases, the initial operation should be directed towards the treatment of perforation and peritonitis. After the recovery of the patient and the histological confirmation of the malignancy, an adequate staging can be completed and radical oncological operation for gastric cancer may be planned, if appropriate [**3**]-**5**]. 

The paper presents the case of a giant malignant perforation that required an atypical surgical solution in emergency, due to local morphopathological features.

## Case presentation

A 52-year-old male patient was admitted in emergency in our clinic for acute abdomen and signs of peritonitis: diffuse abdominal pain and abdominal rigidity, ileus, nausea and chills. Anamnesis revealed no significant personal or family medical history, but the patient described a decreased appetite and weight loss of approximately 5 kg in the last 4 weeks.

Paraclinical exams supported the diagnoses of peritonitis, with leukocytosis (18000/ mmc), increased CRP and ESR (20mm/ h). The lab test also showed severe anemia (Hb 7 mg/ dl). As simple abdominal X-ray revealed pneumoperitoneum, an emergency surgery was mandatory.

After midline laparotomy, purulent generalized peritonitis, peritoneal carcinomatosis, ascitis and a giant malignant gastric perforation of 3,5-4 cm diameter were encountered. The surrounding gastric walls were rigid, infiltrated by the neoplastic process, with signs of gastric linitis plastica. The intent to mobilize the stomach was difficult and risky, so the classical techniques for repair could not be applied.

Due to the lack of the available gastric material, an atypical solution of gastric packing with greater omentum by means of transgastric traction had to be used. The intra-surgery images are relevant regarding this aspect.

**Fig. 1A,B:**
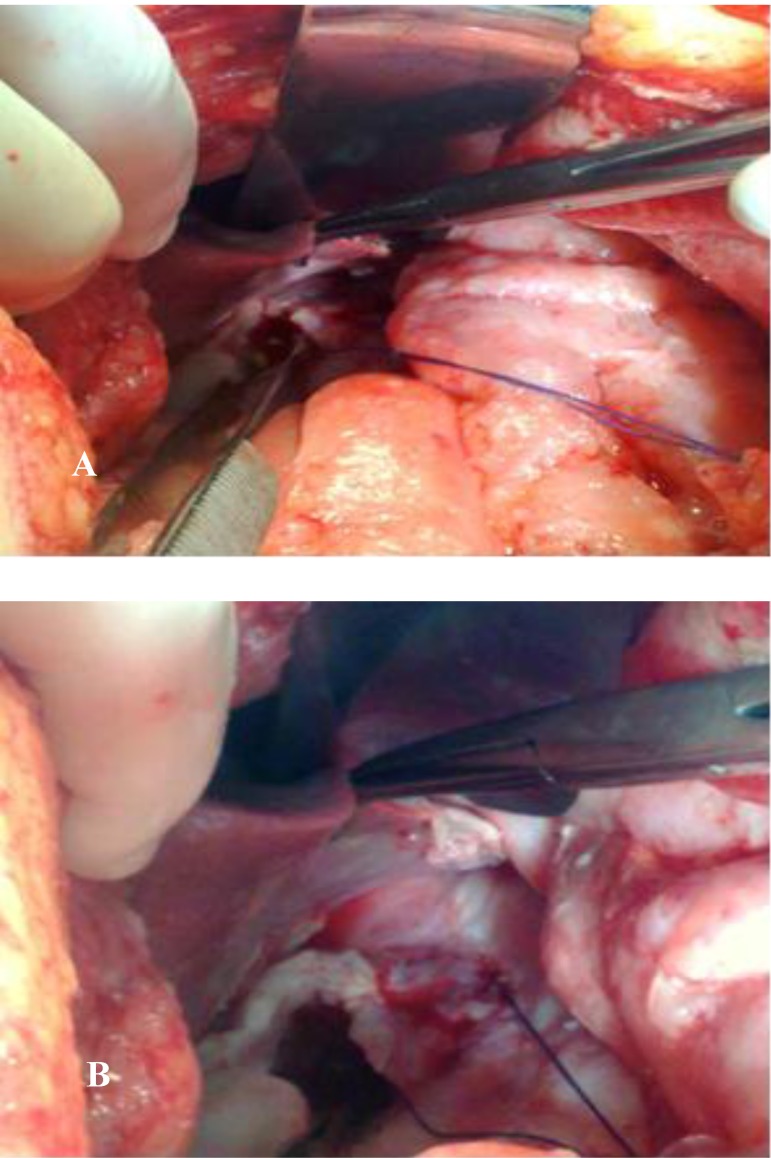
Intraoperatory aspects: giant malignant perforation with anfractuous borders; thickened and rigid gastric walls

**Fig. 2A,B:**
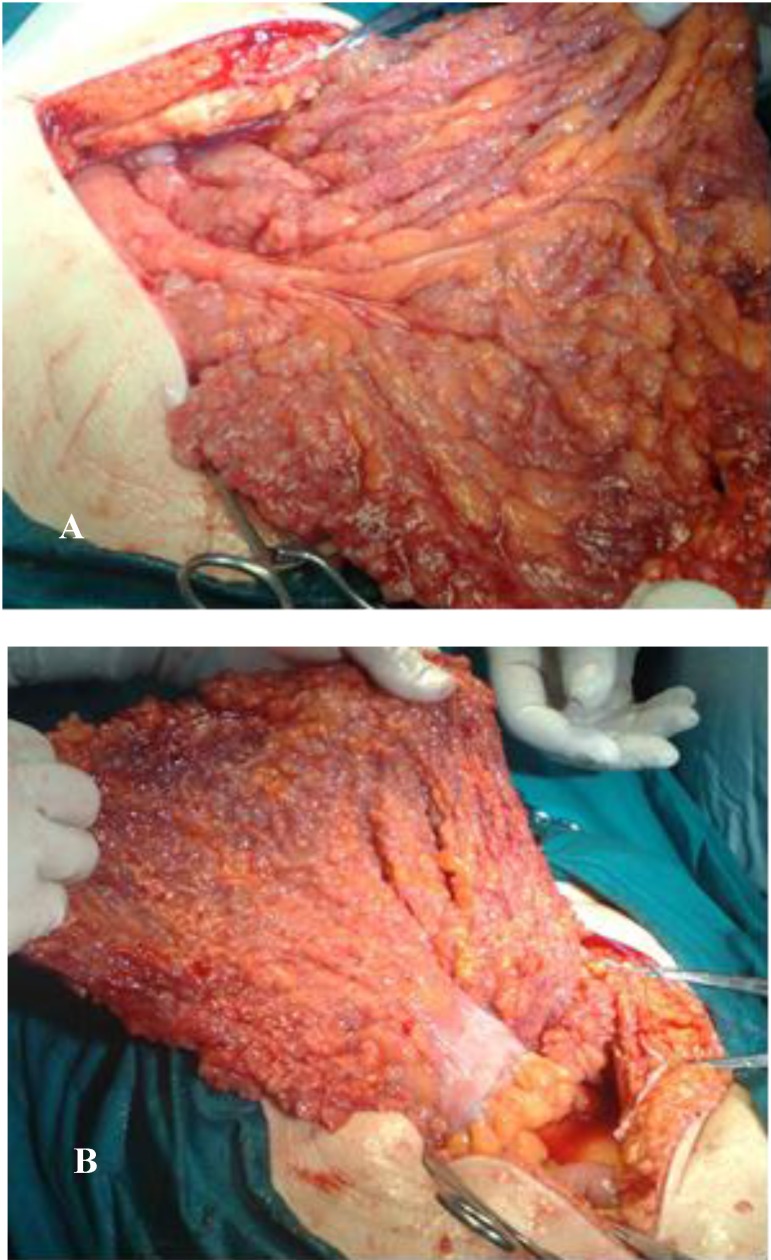
Intraoperatory aspects: greater omentum is used for gastric repair; numerous whitish areas suggestive for mucinous carcinoma metastasis

The surgical technique had to be adapted to the local conditions. It includes highlighting the 3 distinct anatomical areas, individualized and personalized as operatory steps. The greater omentum became the efficient mobile part, which could be adapted for the creation of the contention effect. The gastric part is part of the technical criteria and the imposition carrying transduction becomes omentoplasty. The anatomic oro-gastric path represents the third distinct component reserved for supporting and handling the entire device and tissue-to-tissue fixation, whose biosynthesis is effective in time.

Materials, tools, and devices are specific and are part of the arsenal for transgastric omentoplasty kit.

A fragment of 4/3/1 cm of epiploic tissue was collected and sent to the histopathological examination for confirmation. The macroscopic examination showed whitish areas suggestive of mucinous carcinoma metastasis. The microscopic examination of hematoxylin and eosin-stained tissue sections from the formalin-fixed, paraffin-embedded surgical specimens revealed invasive mucinous carcinoma epiploic metastasis.

**Fig. 3:**
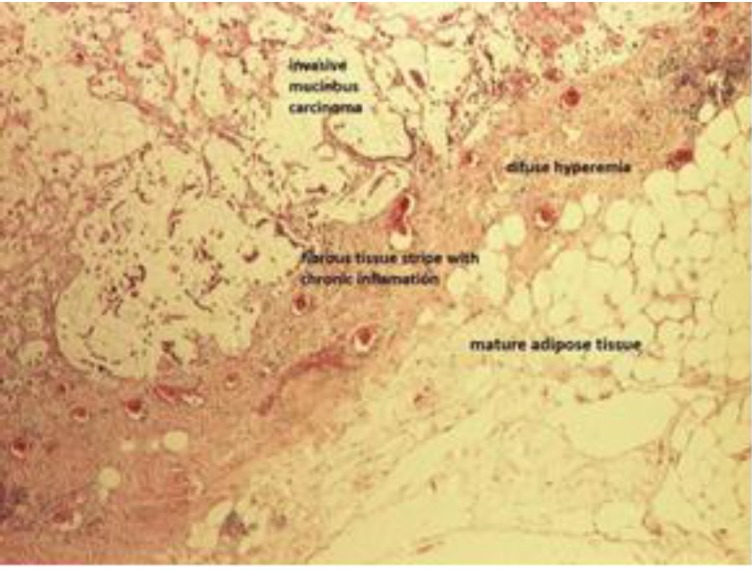
Overall image: hematoxylin and eosin-stained epiploon sections. 4x enlargement: Mature adipose tissue (bottom right), separated from the invasive mucinous carcinoma (top left), by the fibrous tissue stripe with chronic inflammation and diffuse hyperemia (red circles)

**Fig. 4:**
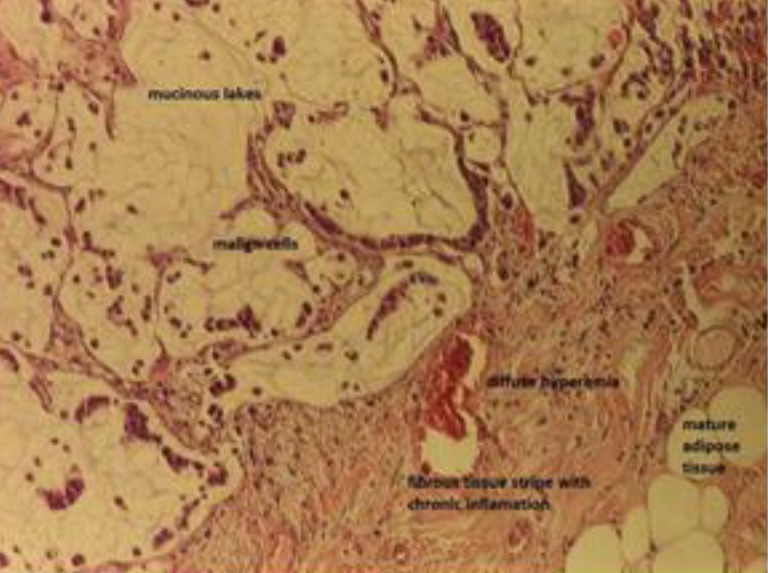
Previous image detail, in order to see the mucinous lakes in which the malign cells float (top left); hematoxylin and eosin-stained sections, 10x enlargement

**Fig. 5:**
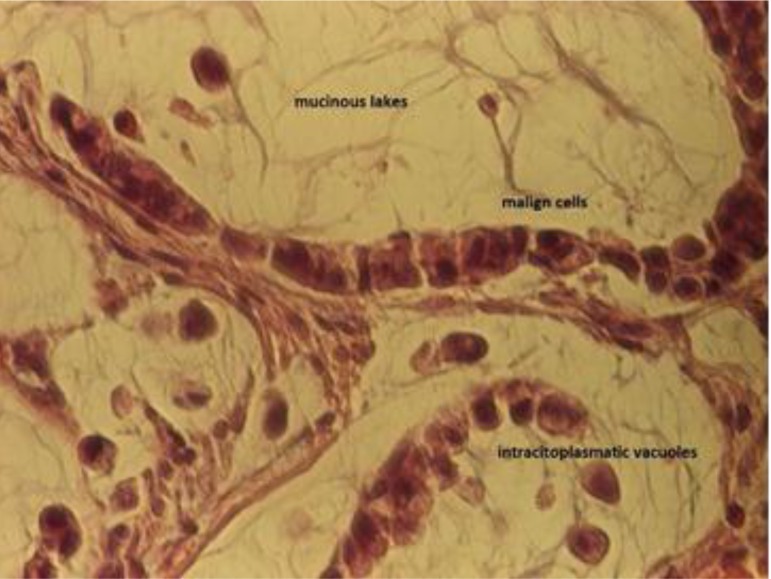
Details to highlight nuclear pleomorphism and the intracytoplasmic vacuoles of this type of carcinoma; hematoxylin and eosin-stained tissue section, 40x enlargement

The postoperative follow up includes both drainage of subhepatic lodge, individualized in this case with two drain tubes (Pezzer and silicone) and a nasogastric probe. Loco-regional specific imagistics is imposed by the initiation of oral food intake.

The Barium radiograph 19 days post-surgery revealed: normal esophagus, increased fornix-diaphragm distance, giant malignant niche, with normal gastro-intestinal transit. Thoracic X-ray showed pleural liquid in both pulmonary bases.

**Fig. 6A,B:**
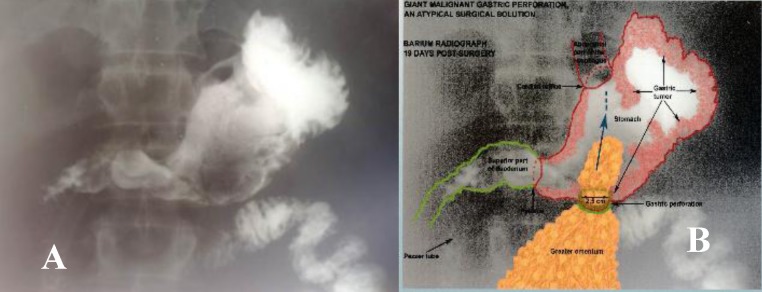
Barium radiograph (A); correlation to local post-surgery anatomical aspect (B)

The discharge diagnosis was perforated mucinous gastric carcinoma, gastric linitis, peritoneal carcinomatosis, neoplastic ascitis, generalized purulent peritonitis, gastric packing with epiploic material, severe secondary anemia, bilateral pleurisy.

The surgical solution proved successful for the immediate development of gastric perforation. Due to the extensive lesions and general risk factors, the two-stage surgery was chosen for the presented case. The underlying condition was not focused on in emergency, the patient being directed to the Oncology Department.

## Discussions

The mucinous gastric carcinoma (MGC) is defined as an adenocarcinoma in which a substantial amount of extracellular mucin (more than 50% of the tumor) is retained within the tumor and it represents only 2% to 6% of all stomach cancers. In a review of literature, MGC is characterized by deeper invasion, more frequent lymph node metastasis, a more advanced pathologic stage at the moment of diagnosis, more frequent lymphatic invasion, and a poorer survival than in NMGC. Though, the poorer survival prognosis is not related with the histopathological features “per se”, but with the late diagnosis [**6**,**7**].

There are several theories that explain the aggressive invasion of the gastric wall. One of them states that the extracellular mucin acts as an infiltrating medium into the surrounding stroma and assists the penetration of deeper layers by tumor cells [**8**]. Papadopoulos et al. proposed that mucin interferes with the inflammatory response and the immunologic recognition of tumor cells [**9**].

On the other hand, this rare type of gastric cancer goes along with increased thickness and rigidity of gastric walls, with a transmural development which involves little gastric mucosa. In a review of literature, we found no previous case report of malignant perforation associated with this particular type of gastric cancer.

## Conclusions

Due to the local anatomopathological aspects, none of the classical curative or palliative techniques could be successfully applied in the presented case. Local intraoperatory findings were surprised by the discrepancy between the anamnesis and the discrete clinical symptoms and, on the other hand, the severity and extension of the gastric lesions: mucinous gastric carcinoma with extensive infiltration of the walls, giant perforation of 3,5/ 4 cm, with anfractuous borders, suggestive of a long term gastric sufferance. The stomach was rigid and difficult to mobilize at the moment and intraoperative risk became prohibitive. Due to rare histopathological encountering (perforation on gastric linitis plastica), an atypical solution had to be chosen, using the greater omentum as material for the surgical repair of the defect [**10**].
